# Ultra-Low-Bubble-Density Quartz Glass Enabled by Stepwise Calcination of High-Purity Synthetic Quartz Powder

**DOI:** 10.3390/nano16140856

**Published:** 2026-07-12

**Authors:** Woo-Guk Lee, Chang-Jin Lee, Ji-Ho Choi, Ji-Hun Kim, Yohan Choi, Tae-Hun Shim, Jinsub Park, Jea-Gun Park

**Affiliations:** 1 Department of Nanoscale Semiconductor Engineering, Hanyang University, Seoul 04763, Republic of Korea; wooguk@hanyang.ac.kr (W.-G.L.); chjinsu@hanyang.ac.kr (Y.C.); jinsubpark@hanyang.ac.kr (J.P.); 2 Advanced Semiconductor Materials & Device Development Center, Hanyang University, Seoul 04763, Republic of Korea; changjin0479@hanyang.ac.kr (C.-J.L.); agger1212@hanyang.ac.kr (J.-H.K.); thshim@hanyang.ac.kr (T.-H.S.); 3 Department of Electronic Engineering, Hanyang University, Seoul 04763, Republic of Korea; choijiho123@hanyang.ac.kr; 4 Samsung Electronics Co., Ltd., Memory Business, Hwaseong-si 18448, Republic of Korea

**Keywords:** synthetic quartz powder, quartz glass, calcination, bubble density, silanol condensation

## Abstract

High-purity synthetic quartz powders are widely used for quartz crucibles and quartzware in semiconductor processes. However, sol–gel-derived synthetic quartz powders contain hydrogen bonds on pore interiors and surfaces, inducing bubble formation during quartz glass fusion. In this study, the removal behavior of hydrogen bonds depending on calcination temperature was investigated by mass reduction, Brunauer–Emmett–Teller (BET) specific surface area, tap density, and Fourier transform infrared (FT-IR) absorbance. Physisorbed and weakly hydrogen-bonded water (~3350 cm^−1^), vicinal/geminal silanol (~3650 cm^−1^), and isolated silanol (~3745 cm^−1^) were removed in the distinct temperature ranges of 200–600 °C, 700–1000 °C, and above 1100 °C, respectively. Based on this removal behavior, a stepwise calcination process at 300 °C for 5 h, 700 °C for 5 h, and 1200 °C for 10 h was designed. This process reduced OH concentration to 3.6 ppm and decreased bubble density to 0.6 bubbles cm^−3^ in fused quartz glass.

## 1. Introduction

High-purity synthetic quartz powders with heavy metallic contamination levels below 100 parts per billion (ppb) have been widely used as coating materials for fused synthetic quartz layers inside natural quartz crucibles for semiconductor Si ingot growth using the Czochralski (CZ) method, as well as for semiconductor process components such as quartz tubes and boats [1,2,3,4]. In particular, natural quartz crucibles coated with fused synthetic quartz powder must be free of bubbles, because bubbles can diffuse from the inner crucible region into the molten Si ingot and thereby readily generate bubbles in the ingot [5,6,7,8,9]. Residual hydrogen bonds in high-purity synthetic quartz powder can induce bubble formation in natural quartz crucibles coated with fused synthetic quartz powder [9,10]. Thus, calcination has been widely employed to remove the residual hydrogen bonds in high-purity synthetic quartz powder. However, the chemistry of the residual hydrogen bonds in high-purity synthetic quartz powder has not yet been clearly reported, although the broad hydrogen bonds adsorbed on the surfaces of colloidal silica powders or silica thin films are known to include physisorbed water, weakly hydrogen-bonded water, and vicinal, geminal, and isolated silanol bonds [11,12]. The calcination temperatures required to remove these hydrogen bonds from silica materials, such as amorphous silica, fused silica powder, precipitated silica, and silica nanoparticles, are known to vary depending on the specific hydrogen-bonded species involved [12,13,14,15]. Beyond these temperature dependencies, the underlying chemistry has also been described: surface silanol groups are known to condense into siloxane (Si–O–Si) bonds with the release of water upon heating, leading to a shrinkage of the associated pore structure [12], and this pore evolution during calcination of porous silica materials has also been numerically modeled in previous studies [16]. However, the calcination-temperature-dependent removal behavior of specific hydrogen bonds in synthetic quartz powder and its direct effect on the resulting bubble formation in fused quartz glass have not been clearly elucidated.

In this study, this removal behavior was therefore directly linked to the resulting bubble formation by evaluating both the calcined powder and the fused quartz glass, and the detailed chemistry of these hydrogen bonds was analyzed by Fourier transform infrared spectroscopy (FT-IR). Similar to the hydrogen bonds adsorbed on the surfaces of colloidal silica powders, physisorbed and weakly hydrogen-bonded water, and vicinal, geminal, and isolated silanol bonds were identified in the FT-IR spectra at 3200–3600, 3650, and 3745 cm^−1^, respectively, corresponding to broad hydrogen bonds adsorbed on the pore interior and the surface of the synthetic quartz powders [13,17,18,19,20]. The temperature-dependent removal behavior of physisorbed water, vicinal/geminal silanol, and isolated silanol bonds was quantitatively investigated by FT-IR, thermogravimetric analysis (TGA), Brunauer–Emmett–Teller (BET), and tap density analyses. Based on this removal behavior, a stepwise calcination process was designed, so that each calcination step corresponded to one of the three temperature ranges, with the expectation that addressing each hydrogen-bonded species within its characteristic removal temperature range would more effectively reduce residual hydrogen bonds than a single high-temperature treatment, and this process was evaluated by fabricating synthetic quartz glass through vacuum fusion of the calcined powders and measuring the resulting OH concentration, bubble diameter, and bubble density.

## 2. Materials and Methods

### 2.1. Materials

Synthetic quartz powders were synthesized via a sol–gel process using fumed silica (SiO_2_, OCI Company Ltd., Seoul, Republic of Korea), potassium hydroxide (KOH, 48 wt%, EUNJINCHEM, Gunsan, Republic of Korea), deionized water, and hydrochloric acid (HCl, 36–38 wt%, Chemitop, Jincheon, Republic of Korea). The detailed synthesis procedure followed our previous report [21]. To investigate the effect of calcination temperature on broad hydrogen bonds, the synthesized quartz powders were individually calcined at 200, 300, 400, 500, 600, 700, 800, 900, 1000, 1100, and 1200 °C for 1 h. Furthermore, to evaluate the relationship between holding time and the absorbance behavior of broad hydrogen bonds in representative temperature regions, additional calcination experiments were carried out at 300, 700, and 1200 °C for 1, 5, and 10 h. Two different calcination processes were applied to the synthetic quartz powders to compare their effects on the OH concentration and bubble formation in the fabricated quartz glass. The first process consisted of one-step calcination at 1000, 1100, and 1200 °C for 1 h, and at 1200 °C for 10 h, whereas the second employed stepwise calcination at 300 °C for 5 h, followed by 700 °C for 5 h, and finally 1200 °C for 1, 5, or 10 h. The calcined powders were placed into a graphite mold with a diameter of 50 mm and subjected to vacuum fusion at 1800 °C under a reduced pressure of approximately 10^−2^ torr with a heating rate of 5 °C/min, as shown in Figure S1. After fusion, the quartz glass samples were cut into specimens with a thickness of 7 mm and dimensions of approximately 2 × 5 cm^2^. The specimens were subsequently polished using a colloidal silica-based chemical mechanical planarization (CMP) slurry for 2 h to enable reliable characterization of OH concentration and bubble density.

### 2.2. Characterization

The particle size distribution and impurity levels of the synthesized quartz powders were characterized using a particle size analyzer (LA-960, HORIBA Scientific Co., Inc., Kyoto, Japan) and inductively coupled plasma mass spectrometry (ICP-MS, Agilent 8800, Agilent Technologies, Santa Clara, CA, USA), respectively. Thermogravimetric analysis (TGA, STA 449F3 Jupiter^®^, Netzsch, Selb, Germany) was performed to evaluate the dehydroxylation behavior of broad hydrogen bonds. The specific surface area of the quartz powders was measured by Brunauer–Emmett–Teller (BET) analysis (3Flex, Micromeritics, Norcross, GA, USA). The tap density of the calcined synthetic quartz powder was measured by placing approximately 1 g of powder into a 5 mL cylinder, which was vibrated using a sieve shaker for 1 min until the powder volume stabilized. The tap density was then calculated as the ratio of the powder mass, measured using a precision balance, to the stabilized packed volume read from the cylinder. The relative OH content in the quartz powders was analyzed by Fourier-transform infrared spectroscopy (FT-IR, Nicolet iS50, Thermo Fisher Scientific Inc., Waltham, MA, USA) in attenuated total reflection (ATR) mode in the wavenumber range of 2500–4000 cm^−1^, with a resolution of 4 cm^−1^ and 64 scans per spectrum, and the absorbance at 3350, 3650, and 3745 cm^−1^ was then extracted to evaluate the removal behavior of physisorbed and weakly hydrogen-bonded water, vicinal/geminal silanol, and isolated silanol bonds, respectively. To evaluate the bubble density and size distribution in quartz glass samples fabricated under different calcination conditions, a visible-light camera-based automatic wafer inspection system adapted for bubble detection and analysis (AWI-12-2L, NEXUS1, Hwasung, Republic of Korea) was employed. The OH concentration of the fused-glass specimen fabricated under each calcination condition was measured at its center using FT-IR. Bubble density was evaluated from six fields of view (14.2 mm × 7.99 mm) per specimen, using a visible-light camera with a spectral response range of 400–800 nm and a minimum detectable bubble size of 10 μm, employing both ring-shaped and backlight illumination. Bubble diameter was determined by measuring up to five bubbles from each field of view; when fewer than five bubbles were detected, all detectable bubbles were measured, and bubble diameter is reported as the number-weighted mean of the measured bubbles. Error bars represent the minimum and maximum values obtained from the measurements.

## 3. Results and Discussion

### 3.1. Dependence of Mass, BET Specific Surface Area, and Tap Density on Calcination Temperature for High-Purity Synthetic Quartz Powder

**Figure 1:**
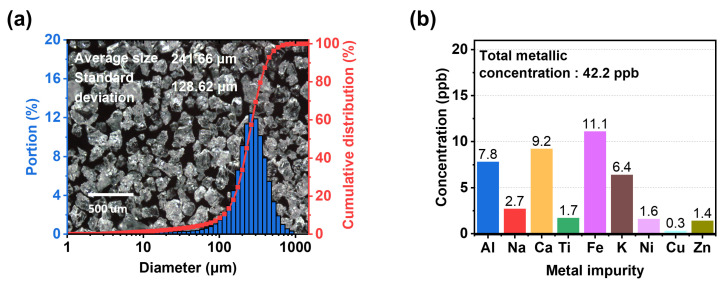
Morphology and metal contamination level of as-synthesized synthetic quartz powders. (**a**) Optical microscopic image and size distribution and (**b**) metallic concentrations for as-synthesized synthetic quartz powders.

High-purity synthetic quartz powders were synthesized using a sol–gel process, as reported in our previous work [21]. The as-synthesized powders exhibited an irregular morphology, with an average powder size of 241.66 μm and a standard deviation of 128.62 μm, and contained numerous internal pores, as shown in Figure 1a. The total metallic concentration was 42.2 ppb, including Al, Na, Ca, Ti, Fe, K, Ni, Cu, and Zn, as shown in Figure 1b. It should be noted that synthetic quartz powders for semiconductor applications require a metallic contamination level of less than 100 ppb.

**Figure 2:**
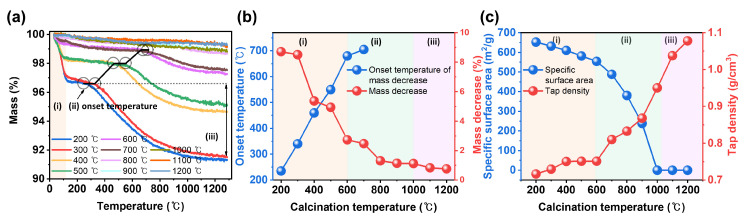
Thermogravimetric analysis (TGA) and Brunauer–Emmett–Teller (BET) analysis of quartz powder calcined at temperatures from 200 to 1200 °C for 1 h. (**a**) TGA curves of mass reduction as a function of TGA temperature for synthetic quartz powders calcined at different calcination temperatures, (**b**) onset temperature of mass decrease and total mass loss of synthetic quartz powders depending on calcination temperature, and (**c**) BET specific surface area and tap density of quartz powder at calcination temperatures from 200 to 1200 °C for 1 h.

The TGA behavior of the calcined high-purity synthetic quartz powders was investigated as a function of calcination temperature, namely 200–1200 °C, where the calcination time was fixed at 1 h, as shown in Figure 2a. For the synthetic quartz powders calcined at 200–600 °C, the mass initially decreased significantly up to the onset temperature in the TGA curves and then decreased gradually with further increases in TGA temperature, as shown in Figure 2a. The onset temperature was defined as the intersection between the tangent line extrapolated from the initial baseline and the tangent line extrapolated from the region of most rapid mass loss in the TGA curve. In contrast, for the synthetic quartz powders calcined above 700 °C, the mass decreased only gradually and slightly with increasing TGA temperature without showing a distinct onset temperature. The onset temperature increased from 235 to 680 °C as the calcination temperature increased from 200 to 600 °C, and then saturated and disappeared at higher calcination temperatures, as shown in region (i) of Figure 2b. In the calcination temperature range of 200–600 °C, the mass decreased remarkably (8.71 to 2.73%), while the BET specific surface area decreased only slightly (652.12 to 554.19 m^2^/g) and the tap density increased from 0.717 to 0.751 g/cm^3^, as shown in Figure 2b,c. In the calcination temperature range of 700–1000 °C, the mass decreased only slightly (2.47 to 1.12%), while the BET specific surface area decreased abruptly (488.04 to 0.14 m^2^/g) and the tap density increased remarkably, as shown in Figure 2b,c, consistent with previously reported large reductions in BET specific surface area upon high-temperature calcination of sol–gel-derived silica materials due to pore collapse [22]. In the calcination temperature range of 1100–1200 °C, the mass, BET specific surface area, and tap density all changed only slightly, as shown in Figure 2b,c. The tap density increased from 0.717 g/cm^3^ at 200 °C to 1.078 g/cm^3^ at 1200 °C, indicating that the internal porosity of the synthetic quartz powder decreased with increasing calcination temperature. In summary, the dependence of the BET specific surface area and tap density on calcination temperature clearly demonstrated three specific calcination temperature ranges, namely 200–600, 700–1000, and 1100–1200 °C, which are associated with the temperature-dependent removal of specific broad hydrogen bonds, namely physisorbed and weakly hydrogen-bonded water, vicinal/geminal silanol, and isolated silanol bonds, respectively.

### 3.2. FT-IR Absorbance Spectra of the Remaining Broad OH Bonds (~3350 cm^−1^), Vicinal/Geminal Silanol Bonds (~3650 cm^−1^), and Isolated Silanol Bonds (~3745 cm^−1^) Depending on Calcination Temperature in High-Purity Synthetic Quartz Powder

**Figure 3:**
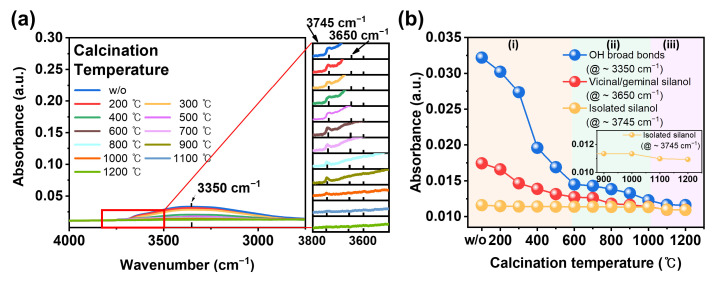
FT-IR spectra of synthetic quartz powders calcined at temperatures from 200 to 1200 °C for 1 h. (**a**) Absorbance spectra in the wavenumber range of 2500–4000 cm^−1^, with enlarged absorbance spectra in the wavenumber range of 3500–3800 cm^−1^, and (**b**) absorbance of OH broad bonds (~3350 cm^−1^), vicinal and geminal silanol bonds (~3650 cm^−1^), and isolated silanol bonds (~3745 cm^−1^) as a function of calcination temperature.

According to previous reports [12,13,19,20], the absorbance in the wavenumber range of 3200–3600 cm^−1^, with a peak centered at approximately 3350 cm^−1^, is assigned to physisorbed and weakly hydrogen-bonded water, referred to here as OH broad bonds. The absorbance at ~3650 cm^−1^ is attributed to vicinal/geminal silanol bonds, and the absorbance at ~3745 cm^−1^ is attributed to isolated silanol bonds, as shown in Figure 3a. In the calcination temperature range of 200–600 °C, the absorbance of the broad OH bonds at 3350 cm^−1^ decreased sharply from 0.03023 to 0.01449, and the absorbance of vicinal/geminal silanol bonds at 3650 cm^−1^ decreased considerably from 0.01659 to 0.01272, as shown in Figure 3b, while the mass decreased remarkably and the BET specific surface area decreased only slightly, as shown in Figure 2b,c. This indicates that calcination at 200–600 °C mainly eliminates physisorbed and weakly hydrogen-bonded water adsorbed within the pore interiors and on the surfaces of the synthetic quartz powders through desorption and out-diffusion of water gas. In the calcination temperature range of 700–1000 °C, the absorbance at 3350 cm^−1^ decreased noticeably from 0.01429 to 0.01228, and the absorbance at 3650 cm^−1^ decreased slightly from 0.01260 to 0.01138, as shown in Figure 3b, coinciding with the region where the BET specific surface area decreased abruptly from 488.04 to 0.14 m^2^/g, as shown in Figure 2c. This indicates that calcination at 700–1000 °C principally removes vicinal/geminal silanol bonds adsorbed within the pore interiors and on the surfaces of the synthetic quartz powders through silanol condensation, resulting in the production of siloxane bonds, namely Si–O–Si, the out-diffusion of water gas, and shrinkage of the internal pore structure of the calcined synthetic quartz powders. In the calcination temperature range of 1100–1200 °C, the absorbance at 3350 cm^−1^ decreased only slightly from 0.01164 to 0.01161, and the absorbance at 3650 cm^−1^ decreased only marginally from 0.01096 to 0.01095, while the absorbance of isolated silanol bonds at 3745 cm^−1^, which had remained nearly constant at approximately 0.01135 up to 1000 °C, decreased very slightly, as shown in the inset of Figure 3b, consistent with the mass, BET specific surface area, and tap density all changing only slightly in this range, as shown in Figure 2b,c. This indicates that calcination at 1100–1200 °C dominantly removes isolated silanol bonds adsorbed within the pore interiors and on the surfaces of the synthetic quartz powders.

### 3.3. Removal Efficiencies of the Broad OH Bonds, Vicinal/Geminal Silanol, and Isolated Silanol Bonds Depending on Calcination Temperature and Holding Time in High-Purity Synthetic Quartz Powder

**Figure 4:**
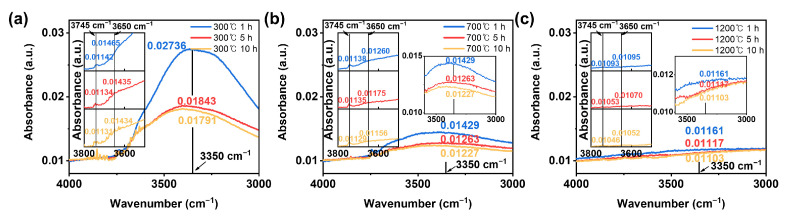
FT-IR absorbance spectra of synthetic quartz powders calcined at 300, 700, and 1200 °C for 1, 5, and 10 h. (**a**) 300 °C, (**b**) 700 °C, and (**c**) 1200 °C. In the inset, magnified FT-IR spectra highlighting the absorbance of OH broad bonds (~3350 cm^−1^), vicinal/geminal silanol bonds (~3650 cm^−1^), and isolated silanol bonds (~3745 cm^−1^).

To investigate the effect of calcination holding time on the removal of the broad OH bonds, vicinal/geminal silanol, and isolated silanol bonds, three representative temperatures, namely 300, 700, and 1200 °C, were selected, and the FT-IR absorbance spectra at these temperatures were examined as a function of holding time, as shown in Figure 4 and Table S1. For the synthetic quartz powder calcined at 300 °C, the absorbance at 3350 cm^−1^ decreased considerably from 0.02736 to 0.01843 as the holding time increased from 1 to 5 h, and then decreased slightly to 0.01791 at 10 h, corresponding to a reduction of approximately 34.6%, as shown in Figure S2a. This result indicates that physisorbed and weakly hydrogen-bonded water are dominantly removed at 300 °C, and that most of these bonds are eliminated within approximately 5 h, as shown in Figure 4a. In contrast, the absorbance at 3650 cm^−1^ and at 3745 cm^−1^ decreased only slightly and nearly linearly from 0.01465 to 0.01434 and from 0.01142 to 0.01131, respectively, as the holding time increased from 1 to 10 h. These changes correspond to reductions of 2.2 and 1.0%, respectively, as shown in the inset of Figure 4a and Figure S2a. Therefore, calcination at 300 °C was highly effective in removing physisorbed and weakly hydrogen-bonded water adsorbed within the pore interiors and on the surfaces of the synthetic quartz powders, whereas it was ineffective in removing both vicinal/geminal and isolated silanol bonds, as shown in Figure S2a.

For the synthetic quartz powder calcined at 700 °C, the absorbance at 3350 cm^−1^ and at 3650 cm^−1^ decreased considerably from 0.01429 to 0.01263 and from 0.01260 to 0.01175, respectively, as the holding time increased from 1 to 5 h, as shown in Figure 4b and Figure S2b. Thereafter, they decreased only marginally from 0.01263 to 0.01227 and from 0.01175 to 0.01156, respectively, as the holding time increased from 5 to 10 h. These changes correspond to reductions of 14.2 and 8.3%, respectively, as shown in the left and right insets of Figure 4b and Figure S2b. In contrast, the absorbance at 3745 cm^−1^ decreased only slightly and nearly linearly from 0.01138 to 0.01129, corresponding to a reduction of 0.8%, as the holding time increased from 1 to 10 h, as shown in the left inset of Figure 4b and Figure S2b. Comparing Figure S2a,b, the absorbance at both 3350 cm^−1^ and 3650 cm^−1^ decreased significantly when the calcination temperature increased from 300 to 700 °C. This is because calcination at 700 °C includes the heating period from 300 to 700 °C during ramping, which was approximately 80 min. In particular, calcination at 700 °C was highly effective in removing vicinal/geminal silanol bonds located within the pore interiors and on the surfaces of the synthetic quartz powders, whereas calcination at 300 °C did not noticeably reduce the vicinal/geminal silanol bonds.

Furthermore, for the synthetic quartz powder calcined at 1200 °C, the absorbance at 3350 cm^−1^ and at 3650 cm^−1^ decreased negligibly from 0.01161 to 0.01103 and from 0.01095 to 0.01052, respectively, as the holding time increased from 1 to 10 h. These changes correspond to reductions of 5.0 and 3.9%, respectively, as shown in the left and right insets of Figure 4c and Figure S2c. In contrast, the absorbance at 3745 cm^−1^ decreased significantly from 0.01093 to 0.01053 as the holding time increased from 1 to 5 h, and then decreased slightly from 0.01053 to 0.01046 as the holding time increased from 5 to 10 h, corresponding to a total reduction of 4.3%, as shown in the left inset of Figure 4c and Figure S2c. Comparing Figure 4b,c, calcination at 1200 °C was evidently effective in eliminating isolated silanol bonds located within the pore interiors and on the surfaces of the synthetic quartz powders, rather than in removing the broad OH bonds or vicinal/geminal silanol bonds, consistent with the temperature-dependent trend inferred from the correlation between the FT-IR absorbance, BET specific surface area, and tap density.

### 3.4. Effect of the Stepwise Calcination Process on Reducing Residual OH Concentration, Bubble Density, and Bubble Diameter in Quartz Glass Vacuum-Fused from High-Purity Synthetic Quartz Powder

**Figure 5:**
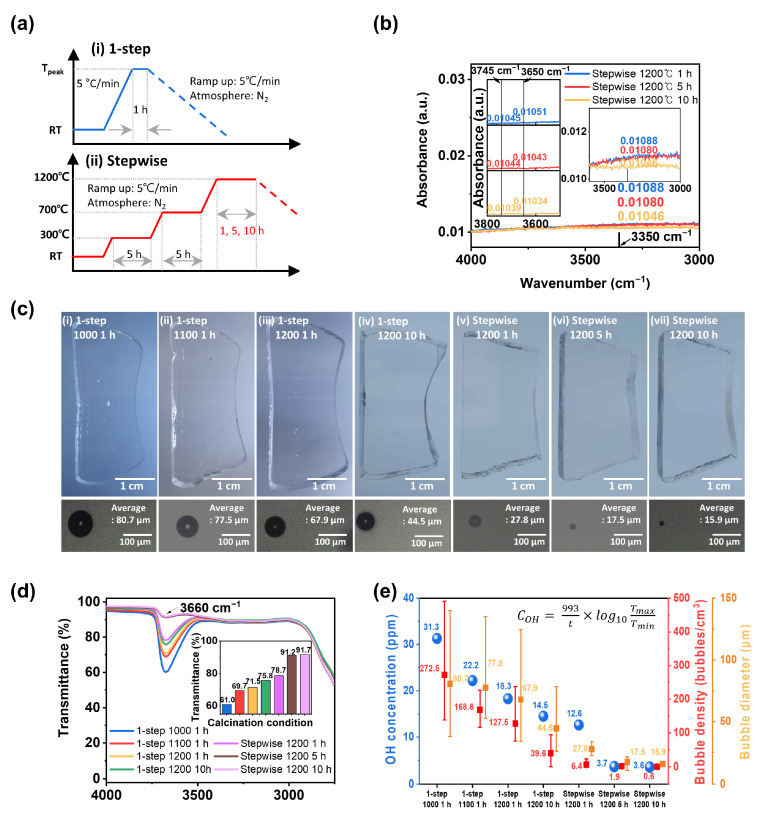
Dependence of bubble diameter and density on calcination process for quartz glass fused by synthetic quartz powders. (**a**) Schematic illustrations of the calcination processes for synthetic quartz powder: (i) one-step calcination and (ii) stepwise calcination. (**b**) FT-IR absorbance spectra of synthetic quartz powders after stepwise calcination followed by 300 °C for 5 h, 700 °C for 5 h, and at 1200 °C for 1, 5, and 10 h. The magnified spectra absorbance (insets) of OH broad bonds (3350 cm^−1^), vicinal/geminal silanol bonds (3650 cm^−1^) and isolated silanol bonds (3745 cm^−1^). (**c**) Optical microscopic images of quartz glass and representative bubble images of each quartz glass. (**d**) FT-IR transmittance spectra of quartz glass fused by calcined synthetic quartz powders. (**e**) OH concentration, bubble density, and bubble diameter of quartz glass fabricated by one-step and stepwise calcination processes.

Based on the removal efficiencies of the broad OH bonds, vicinal/geminal silanol, and isolated silanol bonds as a function of calcination temperature and holding time, as shown in Figure 4 and Figure S2, a stepwise calcination process was designed to maximize the removal of broad hydrogen bonds adsorbed within the pore interiors and on the surfaces of the synthetic quartz powders. The designed process consisted of holding at 300 °C for 5 h, followed by holding at 700 °C for 5 h and then at 1200 °C for 1, 5, or 10 h, as shown in (ii) of Figure 5a. In contrast, the one-step calcination process used in Figure 2, Figure 3 and Figure 4 involved direct ramping to the target temperature, as shown in (i) of Figure 5a. As discussed above, calcination at 300 and 700 °C required a holding time of approximately 5 h to effectively remove the broad OH bonds and vicinal/geminal silanol bonds, respectively; therefore, a holding time of 5 h was selected for both temperatures. The holding time at 1200 °C was varied from 1 to 5 and 10 h to evaluate the removal behavior of isolated silanol bonds, because the absorbance corresponding to isolated silanol bonds continued to decrease slightly with increasing holding time. The removal efficiency of the designed stepwise calcination process was compared with that of the conventional one-step calcination process with holding times of 1, 5, and 10 h, as shown in Figure 4c and Figure 5b. At a holding time of 1 h, the absorbance of the broad OH bonds, vicinal/geminal silanol, and isolated silanol bonds after stepwise calcination were 0.01088, 0.01051, and 0.01045, respectively. These absorbances were lower than those obtained after one-step calcination, namely 0.01161, 0.01095, and 0.01093, by 6.3, 4.1, and 4.4%, respectively. At a holding time of 5 h, the absorbance of the broad OH bonds, vicinal/geminal silanol, and isolated silanol bonds after stepwise calcination were 0.01080, 0.01043, and 0.01044, respectively, which were lower than those after one-step calcination, namely 0.01117, 0.01070, and 0.01053, by 3.4, 2.6, and 0.9%, respectively. Furthermore, at a holding time of 10 h, the absorbance of the broad OH bonds, vicinal/geminal silanol, and isolated silanol bonds after stepwise calcination were 0.01046, 0.01034, and 0.01039, respectively. These absorbances were lower than those obtained after one-step calcination, namely 0.01103, 0.01052, and 0.01046, by 5.2, 1.8, and 0.7%, respectively. These results indicate that the stepwise calcination process clearly enhances the removal efficiency of all broad hydrogen bonds, as further shown in Figure S2d, which summarizes the holding-time dependence of all three absorbance under stepwise calcination conditions. To verify these results, the OH concentration, as well as the diameter and density of bubbles induced by residual hydrogen bonds in the synthetic quartz powders, was evaluated by fabricating synthetic quartz glasses through vacuum fusion of the calcined synthetic quartz powders in a graphite mold, as shown in Figure S1. Optical microscopy images of the synthetic quartz glasses prepared by one-step calcination at 1000, 1100, and 1200 °C for 1 h, by one-step calcination at 1200 °C for 10 h and by stepwise calcination with holding times of 1, 5, and 10 h showed that all the glasses were optically transparent, as shown in Figure 5c. However, the diameter of the residual bubbles in the synthetic quartz glasses decreased from 80.7 to 67.9 μm as the calcination temperature increased from 1000 to 1200 °C in the one-step calcination process. Moreover, it decreased from 67.9 to 44.5 μm as the calcination time increased from 1 to 10 h at 1200 °C in the one-step calcination process. In contrast, it decreased from 27.8 to 15.9 μm as the holding time increased from 1 to 10 h in the stepwise calcination process, as shown in Figure 5c.

The dependence of FT-IR transmittance at 3660 cm^−1^ on the holding time of stepwise calcination, namely 300 °C for 5 h, followed by 700 °C for 5 h, and 1200 °C for 1, 5, or 10 h, was evaluated and compared with that of one-step calcination at 1000, 1100, and 1200 °C for 1 h, as shown in Figure 5d. The FT-IR transmittance at 3660 cm^−1^ reflects the amount of residual hydrogen bonds in the quartz glass. For the one-step calcination process with a holding time of 1 h, the transmittance at 1000, 1100, and 1200 °C was 61.0, 69.7, and 71.5%, respectively, indicating that the amount of residual hydrogen bonds decreased significantly with increasing calcination temperature, as shown in the inset of Figure 5d. Moreover, the transmittance at 1200 °C for 10 h was 75.8%, which was higher than that at 1200 for 1 h (i.e., 71.5%). In addition, the transmittance for the stepwise calcination process having 300 for 5 h + 700 °C for 5h + 1200 °C for 1 h was 78.7% which was higher than that for the calcination at 1200 °C for 10 h (i.e., 75.8%). Thus, the stepwise calcination process having 300 for 5 h + 700 °C for 5h + 1200 °C for 1 h presented better removal performance of the amount of residual OH related groups. In addition, for the stepwise calcination process, the transmittance for holding times of 1, 5, and 10 h at 1200 °C was 78.7, 91.2, and 91.7%, respectively, implying that the amount of residual OH related groups decreased markedly with increasing holding time, as shown in the inset of Figure 5d. The transmittance of the synthetic quartz glass prepared by stepwise calcination with a holding time of 10 h, 91.7%, was approximately 1.5 times higher than that obtained by one-step calcination at 1000 °C, 61.0%.

For further statistical analysis, the OH concentration, average bubble diameter, and bubble density were investigated as a function of calcination condition, as shown in Figure 5e. The OH concentration in the quartz glass was quantitatively determined from the FT-IR transmittance spectra by measuring the transmittance at the baseline wavenumber and at 3660 cm^−1^ and applying Equation (1), derived from the Beer–Lambert law [23,24,25].

(1) $$C_{OH}=\frac{993}{t}\times {log}_{10}\frac{T_{max}}{T_{min}}$$

where *C_OH_* represents the OH concentration (ppm), *t* (mm) is the thickness of the glass sample, *T_max_* is the transmittance at base wavenumber, and *T_min_* is the transmittance at 3660 cm^−1^ wavenumber. The OH concentration in the synthetic quartz glasses decreased from 31.3 to 18.3 ppm as the calcination temperature increased from 1000 to 1200 °C in the one-step calcination process, and further decreased to 14.5 ppm as the calcination time increased to 10 h at 1200 °C, whereas it decreased from 12.6 to 3.6 ppm as the holding time increased from 1 to 10 h in the stepwise calcination process. The average bubble diameter and bubble density in the synthetic quartz glasses decreased from 80.7 μm and 272.5 bubbles/cm^3^ to 67.9 μm and 127.5 bubbles/cm^3^, respectively, as the calcination temperature increased from 1000 to 1200 °C in the one-step calcination process, and further decreased to 44.5 μm and 39.6 bubbles/cm^3^, respectively, as the holding time at 1200 °C increased from 1 to 10 h. In contrast, they decreased from 27.8 μm and 6.4 bubbles/cm^3^ to 15.9 μm and 0.6 bubbles/cm^3^, respectively, as the holding time increased from 1 to 10 h in the stepwise calcination process.

These results demonstrate that the designed stepwise calcination process is highly effective in minimizing both the diameter and density of residual bubbles in synthetic quartz glass compared with the conventional one-step calcination process. To further support the proposed mechanism, for calcined synthetic quartz powders at 200–1200 °C for 1 h, the BET specific surface area and tap density were correlated with the FT-IR absorbance of the three hydrogen-bonded species (OH broad bonds, vicinal/geminal silanol, and isolated silanol bonds), as shown in Figure S3a–c. The impact on the decrease in BET specific surface area and the increase in tap density was largest for isolated silanol, followed by vicinal/geminal silanol, and smallest for OH broad bonds. In addition, for the quartz glass fused from one-step calcined powders (1000, 1100, and 1200 °C for 1 h) and stepwise calcined powders (300 °C for 5 h and 700 °C for 5 h, followed by 1200 °C for 1, 5, or 10 h), the OH concentration and bubble density were correlated with the FT-IR absorbance of the three hydrogen-bonded species in the corresponding calcined synthetic quartz powders, as shown in Figure S3d–f. The impact on the reduction in the OH concentration and bubble density for the fused quartz glasses was likewise largest for isolated silanol, followed by vicinal/geminal silanol, and smallest for OH broad bonds. These results indicate that the remaining hydrogen-bonded species in the calcined synthetic quartz powders directly increased the OH concentration and bubble density in the fused quartz glasses; particularly, the remaining isolated silanol bonds increased the OH concentration and bubble density more than the vicinal/geminal silanol and OH broad bonds.

### 3.5. Mechanism of Broad Hydrogen Bonds Elimination During the Calcination of Synthetic Quartz Powder

**Figure 6:**
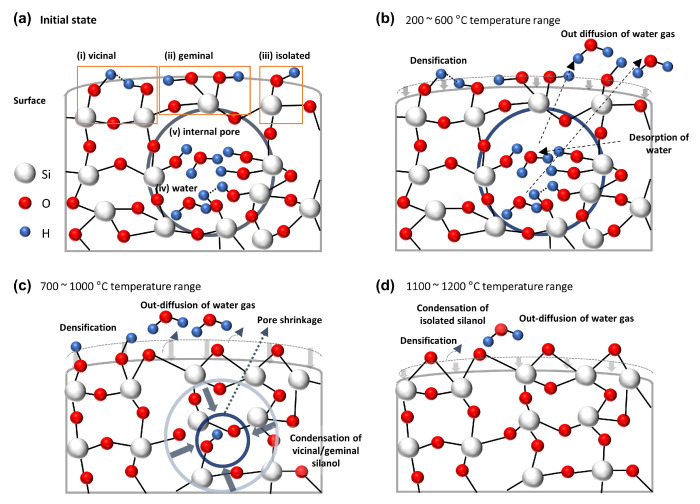
Schematic illustration of the structural characteristics of calcined synthetic quartz powders and its evolution during calcination. (**a**) Initial state of synthetic quartz powder without calcination, showing broad hydrogen bonds distributed on the external surface and internal pore surfaces, including (i) vicinal silanol, (ii) geminal silanol, (iii) isolated silanol, (iv) physisorbed and weakly hydrogen-bonded water, and (v) internal pore. Structural changes in (**b**) the 200–600 °C, (**c**) the 700–1000 °C, and (**d**) the 1100–1200 °C calcination temperature range. The corresponding structural changes, including desorption, condensation, and out-diffusion of water/silanol, for each temperature range are described in detail.

Broad hydrogen bonds, such as physisorbed water, weakly hydrogen-bonded water, vicinal/geminal silanol, and isolated silanol bonds, are adsorbed within the pore interiors and on the surfaces of the synthetic quartz powder after sol–gel synthesis, as shown in Figure 6a. For calcination at 200–600 °C, physisorbed and weakly hydrogen-bonded water adsorbed within the pore interiors and on the surfaces of the synthetic quartz powder were eliminated through desorption and out-diffusion as water gas, as shown in Figure 6b.

In particular, to remove physisorbed and weakly hydrogen-bonded water from the synthetic quartz powder, a higher calcination temperature, namely 200–600 °C, is essentially required compared with silica, fused silica powder, precipitated silica, and silica nanoparticle powders, for which temperatures below 200 °C are generally sufficient, as shown in Table 1. This difference arises because physisorbed and weakly hydrogen-bonded water are adsorbed not only on the external surface but also within the pore interiors of the synthetic quartz powder. It should be noted that the desorption and out-diffusion of physisorbed and weakly hydrogen-bonded water from the pore interiors require higher energy than those from the external surface of the synthetic quartz powder. Therefore, the out-diffusion of physisorbed and weakly hydrogen-bonded water from the pore interiors of the synthetic quartz powders markedly reduced the mass of the synthetic quartz powders, while only moderately decreasing the BET specific surface area without significant condensation between silanol groups, as shown in region (i) of Figure 2b,c. For calcination at 700–1000 °C, vicinal and geminal silanol bonds adsorbed within the pore interiors and on the surfaces of the synthetic quartz powders are condensed, followed by the formation of siloxane bonds (Si–O–Si), and the out-diffusion of water gas, as shown in Equation (2). This process leads to shrinkage of the synthetic quartz powder structure, as shown in Figure 6c.

(2) $$\equiv Si-OH+\equiv Si-OH\to \equiv Si-O-Si\equiv +H_{2}O\;(g)$$

Similar to the removal of physisorbed and weakly hydrogen-bonded water from the synthetic quartz powders, a higher energy is required to eliminate vicinal/geminal silanol bonds from the pore interiors than from the external surface. Therefore, a higher calcination temperature, namely 700–1000 °C, is required for the synthetic quartz powders compared with silica, fused silica powder, precipitated silica, and silica nanoparticle powders, for which temperatures of 200–900 °C are generally sufficient, as shown in Table 1. It should also be noted that vicinal/geminal silanol bonds are mainly adsorbed on the surfaces of fused silica powder, precipitated silica, and silica nanoparticle powders. Thus, the condensation of vicinal/geminal silanol bonds and the out-diffusion of water gas result in a slight reduction in mass and a significant decrease in BET specific surface area, as shown in region (ii) of Figure 2b,c. Furthermore, for calcination at 1100–1200 °C, isolated silanol bonds adsorbed within the pore interiors and on the surfaces of the synthetic quartz powders are removed from both the surface and pore interiors, with limited condensation between isolated silanol bonds, as shown in Figure 6d. As a result, the mass and BET specific surface area of the synthetic quartz powder showed almost no change, as shown in region (iii) of Figure 2b,c. Since higher out-diffusion energy is required for isolated silanol bonds located within the pore interiors than for that located on the external surface of the synthetic quartz powders, a higher calcination temperature, namely 1100–1200 °C, is required to remove isolated silanol bonds from the synthetic quartz powders compared with silica, fused silica powder, precipitated silica, and silica nanoparticle powders, for which temperatures of 900–1200 °C are generally applied, as shown in Table 1.

## 4. Conclusions

The calcination behavior of high-purity synthetic quartz powder revealed that residual OH-related broad hydrogen-bonded species are removed through three distinct temperature ranges. The combined TGA, BET, tap density, and FT-IR analyses showed that physisorbed and weakly hydrogen-bonded water, vicinal/geminal silanol groups, and isolated silanol groups are mainly eliminated at 200–600 °C, 700–1000 °C, and 1100–1200 °C, respectively. Compared with typical amorphous silica surfaces, these removal temperatures are shifted to higher ranges, indicating that a significant fraction of the remaining OH species is located within pore interiors, where removal is more difficult than from external surfaces. Based on this mechanistic understanding, a stepwise calcination process was designed so that each calcination step corresponded to one of the three temperature ranges. Quartz glass fused from powder calcined by this stepwise process showed a residual OH concentration of approximately 3.6 ppm and a bubble density of 0.6 bubbles/cm^3^, with an average bubble diameter of approximately 15.9 μm, compared with approximately 18.3 ppm, 127.5 bubbles/cm^3^, and 67.9 μm, respectively, for the conventional one-step process at 1200 °C for 1 h. Comparing one-step and stepwise calcination under nearly equal total holding times further indicated that the removal of broad OH bonds benefits from the stepwise calcination process itself, beyond what the total thermal exposure alone would predict. Thus, this study suggests a practical calcination strategy for producing low-OH, low-bubble synthetic quartz glass, which serves as a coating material for natural quartz crucibles used in semiconductor silicon ingot growth, as well as in other semiconductor-process quartz glass components. Further optimization of the stepwise temperature range and holding time may enable more complete removal of residual hydrogen bonds adsorbed within the pore interiors of the synthetic quartz powders, further improving compliance with the increasingly strict specifications required for semiconductor-grade applications.

## Figures and Tables

**Table 1:** Comparison of removal temperature ranges for water, vicinal/geminal, and isolated silanol in various silica materials.

Material	Adsorbed Position of Hydrogen Bonds	Removal Temperature Ranges			Ref.
		Water	Vicinal/Geminal Silanol	Isolated Silanol	
Amorphous silica	surface	<~200 °C	~200–900 °C	~900–1200 °C	[12]
Fused silica powder	surface	-	-	>~1100 °C	[13]
Precipitated silica powder	surface	<~200 °C	~200–900 °C	~900–1200 °C	[14]
Silica nanoparticles	surface	<~200 °C	~200–600 °C	>~600 °C	[15]
High-purity synthetic quartz powder	pore interior and surface	~200–600 °C	~700–1000 °C	~1100–1200 °C	This work

## Data Availability

The raw data supporting the conclusions of this article will be made available by the authors on request.

## Supplementary Materials

The following supporting information can be downloaded at https://www.mdpi.com/article/10.3390/nano16140856/s1, Figure S1: Optical images of synthetic quartz powder and quartz glass fused by calcined synthetic quartz powder. (a) Optical images of synthetic quartz powder loaded in a graphite mold, and (b) optical images of quartz glass fused by calcined synthetic quartz powder and a quartz glass cut with a thickness of 7 mm; Table S1: FT-IR absorbance at 3350 cm^−1^, ~3650 cm^−1^, and ~3745 cm^−1^ in wavenumber of synthetic quartz powders calcined at 300, 700, and 1200 °C, and stepwise calcined followed by 300 °C for 5 h, 700 °C for 5 h, and at 1200 °C with holding time of 1, 5, and 10 h. The absorbance at 3350 cm^−1^ corresponding to OH broad bonds, at ~3650 cm^−1^ corresponding to vicinal/geminal silanol bonds, and at ~3745 cm^−1^ corresponding to isolated silanol bonds, presented in Figure 3, Figure 4 and Figure S2a–d; Figure S2: FT-IR absorbance of synthetic quartz powder calcined at 300, 700, and 1200 °C, and stepwise calcined followed by 300 °C for 5 h, 700 °C for 5 h, and at 1200 °C, depending on holding time (i.e., 1, 5, and 10 h). (a) calcination at 300 °C, (b) 700 °C, (c) 1200 °C, and (d) stepwise calcination, being redrawn from Table S1 to understand in detail the dependence of absorbance at ~3350 cm^−1^ (OH broad bonds), ~3650 cm^−1^ (vicinal/geminal silanol bonds), and ~3745 cm^−1^ (isolated silanol bonds) on holding time; Figure S3: Correlation between FT-IR absorbance of hydrogen-bonded species and properties of the synthetic quartz powder and fused quartz glass. Dependence of the specific surface area and tap density on the absorbance at (a) ~3350 cm^−1^ (OH broad bonds), (b) ~3650 cm^−1^ (vicinal/geminal silanol bonds), and (c) ~3745 cm^−1^ (isolated silanol bonds) of one-step calcined synthetic quartz powders (200–1200 °C for 1 h). Dependence of the OH concentration and bubble density on the absorbance at (d) ~3350 cm^−1^, (e) ~3650 cm^−1^, and (f) ~3745 cm^−1^ of quartz glass fused from one-step calcined powders (1000, 1100, and 1200 °C for 1 h) and stepwise calcined powders (300 °C for 5 h and 700 °C for 5 h, followed by 1200 °C for 1, 5, or 10 h).
