# Supplementary material for: Ultra-Low-Bubble-Density Quartz Glass Enabled by Stepwise Calcination of High-Purity Synthetic Quartz Powder
Source: Nanomaterials (Basel). 2026 Jul 12;16(14):856. doi: 10.3390/nano16140856 (PMC13416376; doi:10.3390/nano16140856)
Supplement: Supplementary file 1 [file nanomaterials-16-00856-s001.zip › nanomaterials-4383234-supplementary.pdf]

Supplementary Materials

Ultra-Low-Bubble-Density Quartz Glass Enabled by Stepwise Calcination of High-Purity Synthetic Quartz Powder

Woo-Guk Lee [1,2,†], Chang-Jin Lee [2,3,†], Ji-Ho Choi [3,4], Ji-Hun Kim [2,3], Yohan Choi [1,2], Tae-Hun Shim [2], Jinsub Park [1,3] and Jea-Gun Park [1,2,3,*]

[1] Department of Nanoscale Semiconductor Engineering, Hanyang University, Seoul 04763, Republic of Korea; wooguk@hanyang.ac.kr (W.-G.L.); chjinsu@hanyang.ac.kr (Y.C.); jinsubpark@hanyang.ac.kr (J.P.)
[2] Advanced Semiconductor Materials & Device Development Center, Hanyang University, Seoul 04763, Republic of Korea; changjin0479@hanyang.ac.kr (C.-J.L.); agger1212@hanyang.ac.kr (J.-H.K.); thshim@hanyang.ac.kr (T.-H.S.)
[3] Department of Electronic Engineering, Hanyang University, Seoul 04763, Republic of Korea; choijiho123@hanyang.ac.kr
[4] Samsung Electronics Co., Ltd., Memory Business, Hwaseong-si 18448, Republic of Korea
* Correspondence: parkjgl@hanyang.ac.kr
† These authors contributed equally to this work.

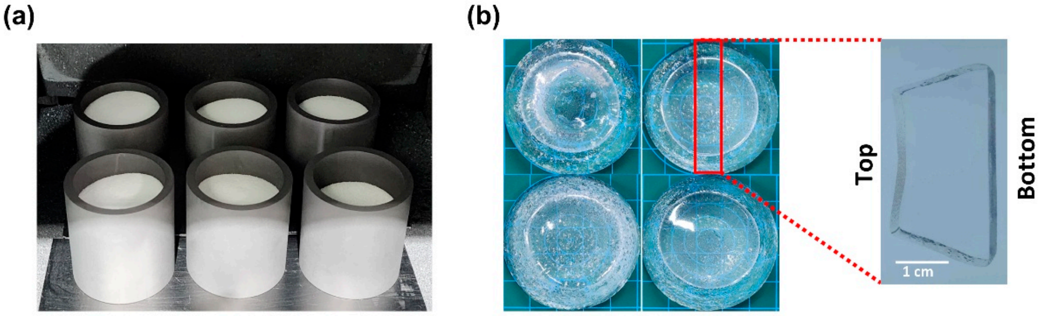

Figure S1. Optical images of synthetic quartz powder and quartz glass fused by calcined synthetic quartz powder. (a) Optical images of synthetic quartz powder loaded in a graphite mold, (b) optical images of quartz glass fused by calcined synthetic quartz powder and a quartz glass cut with a thickness of 7 mm.

Table S1. FT-IR absorbance at 3350 cm^{-1}, ~3650 cm^{-1}, and ~3745 cm^{-1} in wavenumber of synthetic quartz powders calcined at 300, 700, and 1200 °C, and stepwise calcined followed by 300 °C for 5 h, 700 °C for 5 h, and at 1200 °C with holding time of 1, 5, and 10 h. The absorbance at 3350 cm^{-1} corresponding to OH broad bonds, at ~3650 cm^{-1} corresponding to vicinal/geminal silanol bonds, and at ~3745 cm^{-1} corresponding to isolated silanol bonds, presented in Figures 3, 4 and S2(a) –S2(d).

**Calcination Tempera-ture (°C)**	**Holding time (h)**	**Wavenumber**		
		**3350 cm^{-1} (OH broad bonds)**	**3650 cm$^{-1}_{1}$ (vicinal/geminal silanol)**	**3745 cm^{-1} (isolated silanol)**
As-synthesized (Without calcination)	-	0.03220	0.01743	0.01160
	1	0.02736	0.01465	0.01142
300	5	0.01843	0.01435	0.01134
	10	0.01791	0.01434	0.01131
	1	0.01429	0.01260	0.01138
700	5	0.01263	0.01175	0.01135
	10	0.01227	0.01156	0.01129
	1	0.01161	0.01095	0.01093
1200	5	0.01117	0.01070	0.01053
	10	0.01103	0.01052	0.01046
Stepwise calcination (300 °C for 5 h , 700 °C for 5 h , and 1200 °C for 1, 5, 10 h)	1	0.01088	0.01051	0.01045
	5	0.01080	0.01043	0.01044
	10	0.01046	0.01034	0.01039

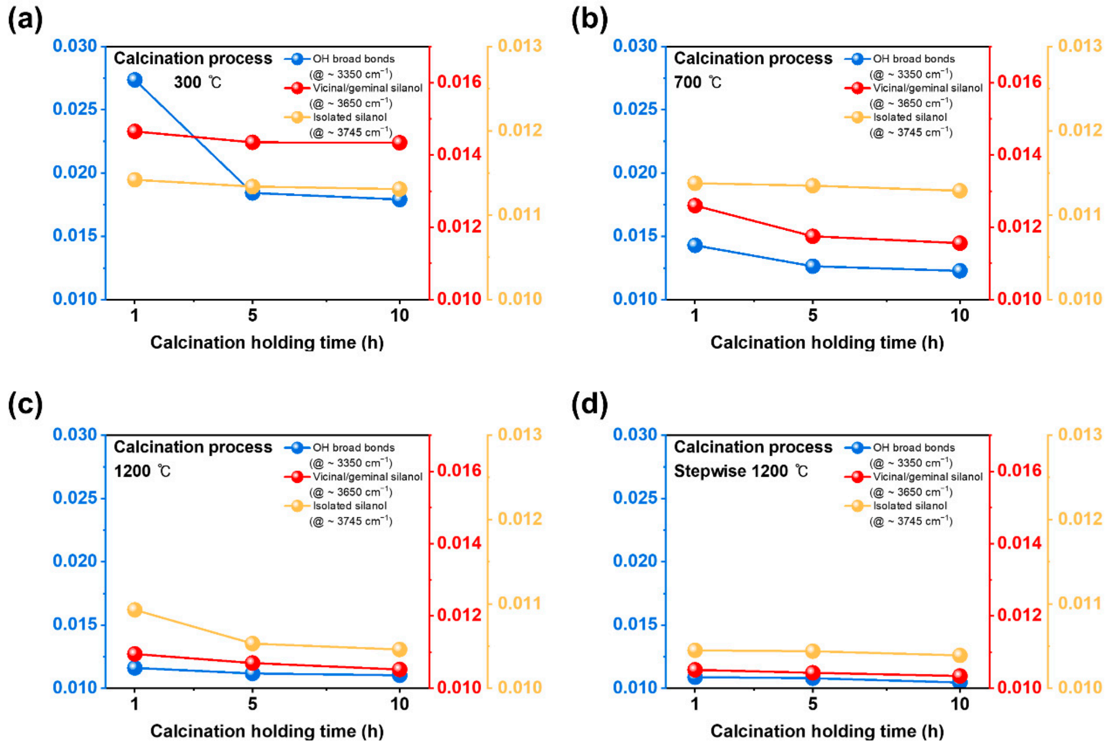

Figure S2. FT-IR absorbance of synthetic quartz powder calcined at 300, 700, and 1200 °C, and stepwise calcined followed by 300 °C for 5 h, 700 °C for 5 h, and at 1200 °C, depending on holding time (i.e., 1, 5, and 10 h). (a) calcination at 300 °C, (b) 700 °C, (c) 1200 °C, and (d) stepwise calcination, being redrawn from Table S1 to understand in detail the dependence of absorbance at ~3350 cm^{-1} (OH broad bonds), ~3650 cm^{-1} (vicinal/geminal silanol bonds), and ~3745 cm^{-1} (isolated silanol bonds) on holding time.

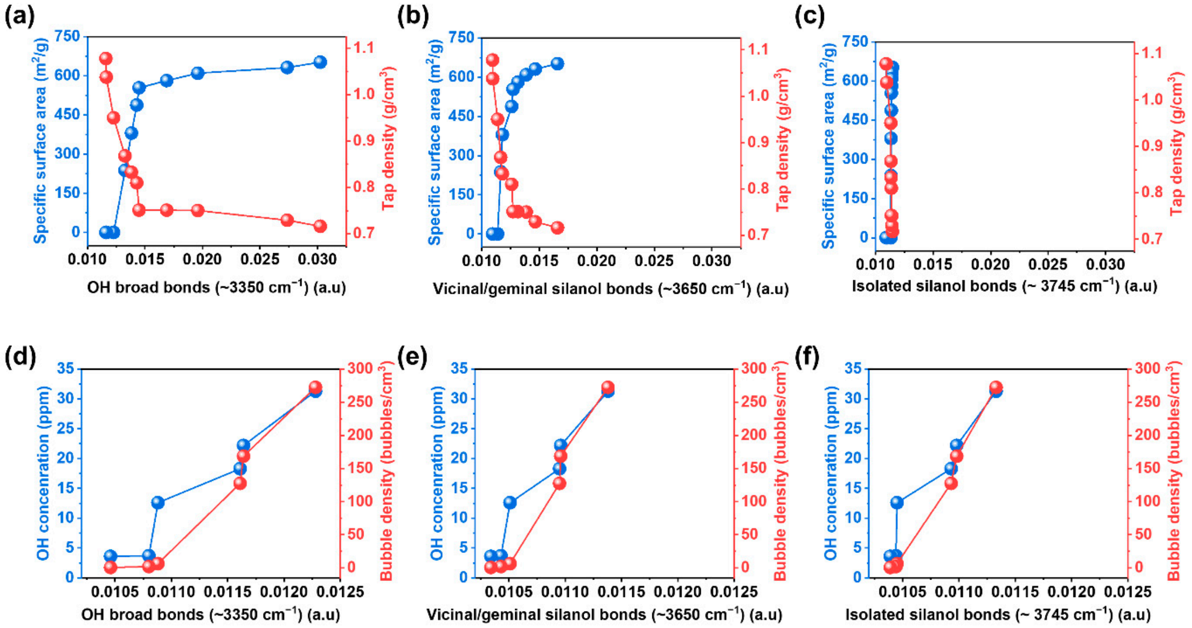

Figure S3. Correlation between FT-IR absorbance of hydrogen-bonded species and properties of the synthetic quartz powder and fused quartz glass. Dependence of the specific surface area and tap density on the absorbance at (a) ~3350 cm^{-1} (OH broad bonds), (b) ~3650 cm^{-1} (vicinal/geminal silanol bonds), and (c) ~3745 cm^{-1} (isolated silanol bonds) of 1-step calcined synthetic quartz powders (200–1200 °C for 1 h). Dependence of the OH concentration and bubble density on the absorbance at (d) ~3350 cm^{-1}, (e) ~3650 cm^{-1}, and (f) ~3745 cm^{-1} of quartz glass fused from 1-step calcined powders (1000, 1100, and 1200 °C for 1 h) and stepwise calcined powders (300 °C for 5 h and 700 °C for 5 h, followed by 1200 °C for 1, 5, or 10 h).
